# Tumor-associated autoantibodies from mouse breast cancer models are found in serum of breast cancer patients

**DOI:** 10.1038/s41523-021-00257-1

**Published:** 2021-05-11

**Authors:** Sasha E. Stanton, Ekram Gad, Erik Ramos, Lauren Corulli, James Annis, Jennifer Childs, Hiroyuki Katayama, Samir Hanash, Jeffrey Marks, Mary L. Disis

**Affiliations:** 1 Cancer Vaccine Institute, University of Washington, Seattle, WA USA; 2 Quellos High Throughput Facility, Institute for Stem Cell and Regenerative Medicine, University of Washington, Seattle, WA USA; 3 Department of Clinical Cancer Prevention, MD Anderson Cancer Center, Houston, TX USA; 4 Division of Surgical Sciences, Duke University, Durham, NC USA

**Keywords:** Breast cancer, Target identification

## Abstract

B cell responses to tumor antigens occur early in breast tumors and may identify immunogenic drivers of tumorigenesis. Sixty-two candidate antigens were identified prior to palpable tumor development in TgMMTV-neu and C3(1)Tag transgenic mouse mammary tumor models. Five antigens (VPS35, ARPC2, SERBP1, KRT8, and PDIA6) were selected because their decreased expression decreased survival in human HER2 positive and triple negative cell lines in a siRNA screen. Vaccination with antigen-specific epitopes, conserved between mouse and human, inhibited tumor growth in both transgenic mouse models. Increased IgG autoantibodies to the antigens were elevated in serum from women with ductal carcinoma in situ (DCIS) and invasive breast cancer (IBC). The autoantibodies differentiated women with DCIS from control with AUC 0.93 (95% CI 0.88–0.98, *p* < 0.0001). The tumor antigens identified early in the development of breast cancer in mouse mammary tumor models were conserved in human disease, and potentially identify early diagnostic markers in human breast tumors.

## Introduction

Breast cancers can be recognized by a patient’s immune system prior to detection by breast imaging with autoantibodies against tumor-associated proteins detected months prior to diagnosis^1,2^. IgG autoantibodies to HER2, p53, CEA, and CYCLIN B1 differentiated women subsequently diagnosed with breast cancer from controls with AUC 0.79 (CI 0.72–0.85, *p* < 0.0001)^3^. Assessment of early human tumor antigens to be used as biomarkers of early detection and vaccine targets, is currently impossible due to breast cancer detection limitations^4^. Antigens from patients with established disease may not be optimal immunologic targets because the tumors have evaded immune surveillance^5,6^. Genetically engineered mouse models that recapitulate human breast cancers may identify antigens at early stages of tumor development, and identifying antigens across multiple mouse models can ensure selecting targets that are expressed across multiple breast cancer subtypes^7,8^. The autoantibody profile in the C3(1)Tag mouse occurs in plasma of women newly diagnosed with triple negative breast cancer^1^, suggesting the utility of mouse models for longitudinal assessment of antigens that occur early during tumor development^9^.

We aimed to identify tumor antigens from autoantibodies identified prior to palpable tumors in two mouse mammary tumor models (TgMMTV-neu and C3(1)Tag), that were involved in survival of HER2 positive and triple negative human breast cancer cell lines as they could be effective immunogens in breast cancer vaccines. We demonstrate that these antigens, identified in a mouse, mediate breast tumor cell survival in HER2 positive and triple negative human cell lines. Autoantibodies against these antigens were found in the serum of women with breast tumors and could be used to discriminate women with high-risk (fibroadenoma and hyperplasia) and pre-invasive (DCIS) breast tumors.

## Results

### Tumor antigens identified in mice prior to palpable tumor are important in survival of HER2 positive and triple negative human breast cancer cell lines

Using two methods, SEREX and natural protein array screens, 63 unique autoantibodies were increased in the serum of TgMMTV-neu and C3(1)Tag mice that would develop breast tumors as compared to FVB parental controls (Supplementary Table 1). There were 35 autoantibodies increased in the TgMMTV-neu mouse model and 33 autoantibodies increased in C3(1)Tag mouse model. Due to overlap in targets recovered, there were 63 unique genes (Supplementary Table 1). All of the candidate proteins had human homologs and 90% (56/63) of the proteins had identified roles in human cancer progression.

**Fig. 1:**
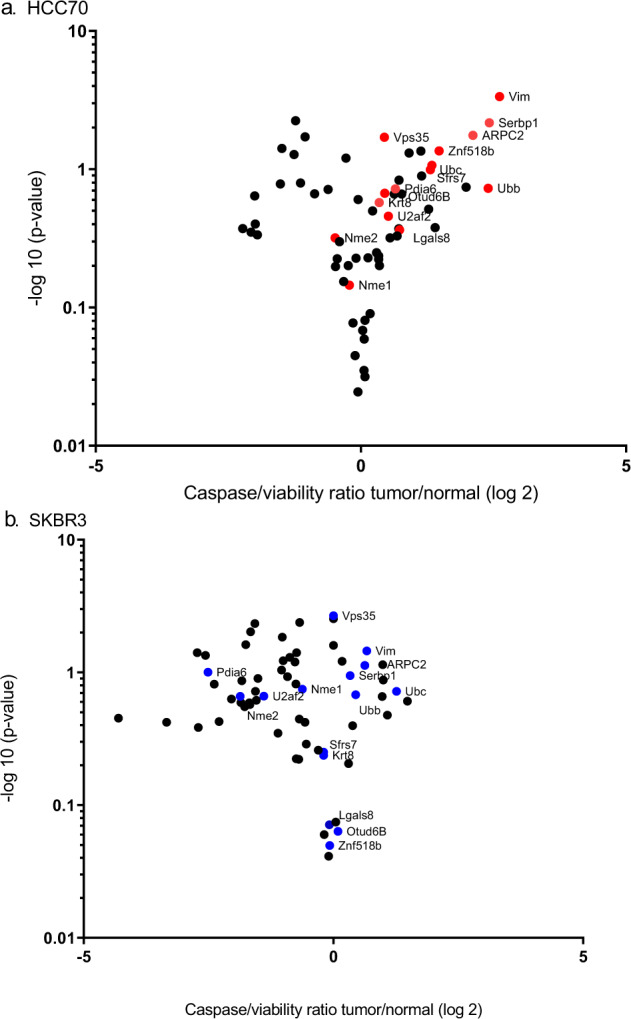
Volcano plots demonstrate siRNA knockdown of the candidate antigens has increased tumor cell line cell death as compared to the normal cell line. Four pooled siRNA were used to knock down expression of each of the sixty-three targets. Evaluation of the apoptosis to cell survival ratio (caspase/viability ratio) in **a** triple negative (red, HCC70), and **b** HER2 positive (blue, SKBR3) human breast cancer cell lines were compared to a non-malignant breast cell line (MCF10F). *Y* axis is –log10 and *X* axis is log2. Targets were selected with higher caspase/cell viability ratio in at least one of the breast cancer cell lines as compared to non-malignant breast cell line (*p* ≤ 0.1).

**Fig. 2:**
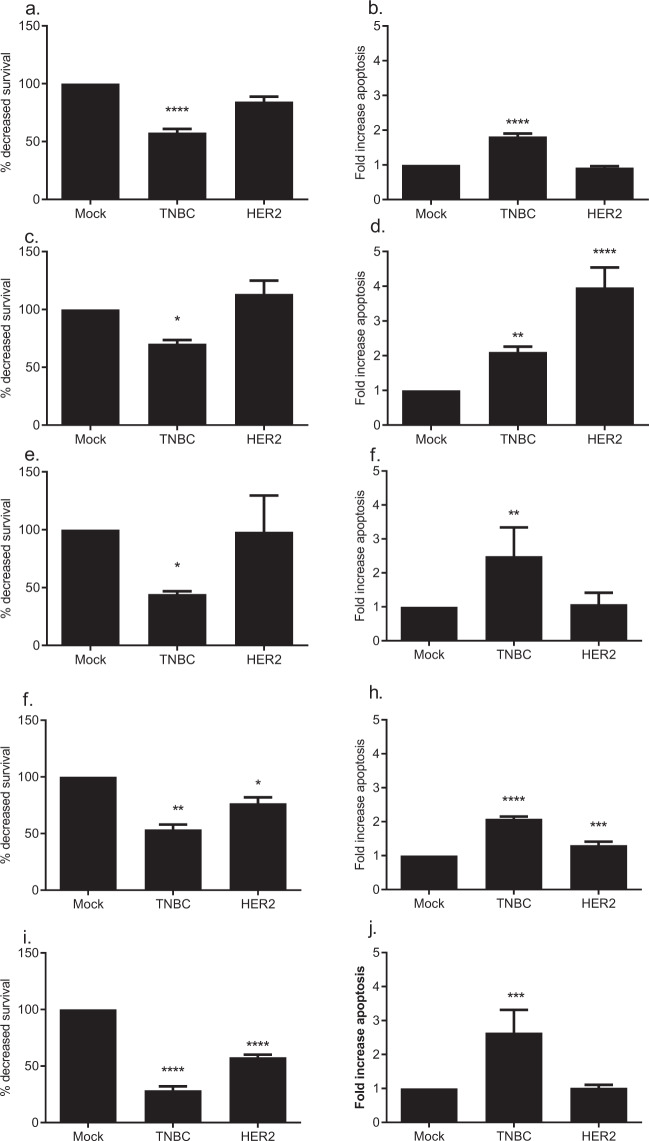
siRNA knockdown of candidate antigen expression decreases cell survival and increases apoptosis in human breast cancer cell lines. Four pooled siRNA knocked down expression of the targets VPS35 (**a**, **b**), ARPC2 (**c**, **d**), SERBP1 (**e**, **f**), KRT8 (**g**, **h**), and Pdia6 (**i**, **j**). Decreased expression of these proteins demonstrate decreased survival (**a**, **c**, **e**, **g**, and **i**) and increased apoptosis (**b**, **d**, **f**, **h**, and **j**) as compared to mock transfected in either triple negative (TNBC, HCC70) or HER2 positive (HER2, SKBR3) human breast cancer cell lines. Survival is measured as % survival compared to mock transfected cells and apoptosis is measured as fold increase compared to mock transfected calls. All studies are in triplicate. Error bars are standard error of mean (SEM) ****p < 0.0001, ***p < 0.001, **p < 0.01, *p < 0.05.

We identified which of the candidate proteins, identified in the mouse, had relevance to survival in human breast cancer^10^. We performed two high-throughput siRNA screens of all 63 candidate antigens in human triple negative (HCC70), and hormone receptor negative HER2 positive (SKBR3) breast cancer cell lines as compared to a non-malignant breast cell line (MCF10F) to identify antigens that were involved in breast tumor growth. We selected SKBR3 cells because, while the TgMMTV-neu mouse is genetically similar to luminal B breast cancer^7^, it is not estrogen driven^11^. Fifteen candidate proteins were recovered from the two unbiased siRNA knockdown screens (ARPC2, KRT8, OTUD6B, SERBP1, SFRS7, UBC, VIM, VPS35, U2AF2, UBB, ZNF518B, NME1, NME2, LGALS8, and PDIA6). siRNA knockdown of all fifteen targets decreased survival in the triple negative cell line and siRNA knockdown of ten of the fifteen decreased survival in the HER2 positive cell line, suggesting that these targets are conserved across these aggressive breast cancer subtypes (Fig. 1). To confirm the high throughput screen, the pooled siRNAs for each of the fifteen proteins were deconvoluted, (1) to demonstrate that each siRNA in the pool specifically knock down the target gene of interest but not a non-specific target by RT PCR (Supplementary Fig. 1), and (2) to confirm the decreased survival and increased apoptosis for each of the four siRNA for each of the candidate proteins in either the HCC70 or SKBR3 breast cancer cell lines (Fig. 2). siRNA knockdown of five of the fifteen proteins (ARPC2, KRT8, SERBP1, VPS35, and PDIA6) confirmed decreased survival and increased apoptosis from the high throughput screens and were conserved between mouse and human (Table 1). In the small scale screens evaluating pooled siRNA, loss of expression of VPS35 decreased survival by 42.3% (*p* < 0.0001) in HCC70 and modestly (15.5%, *p* = 0.06) in SKBR3 and increased apoptosis by 1.8-fold (*p* < 0.0001) in HCC70 (Fig. 2a, b). Loss of expression of ARPC2 decreased survival by 29.6% (*p* = 0.04) in HCC70, but not in SKBR3 and increased apoptosis by 2.1-fold (*p* = 0.005) in HCC70 and 4.0-fold in SKBR3 (*p* < 0.0001) (Fig. 2c, d). Loss of expression of SERBP1 decreased survival by 55.6% (*p* = 0.03) and increased apoptosis by 2.5-fold (*p* = 0.009) in HCC70 (Fig. 2e, f). Loss of expression of KRT8 decreased survival by 46.4% (*p* = 0.0002) in HCC70 and by 23.4% (*p* = 0.04) in SKBR3, and increased apoptosis by 2.1-fold (*p* < 0.0001) in HCC70 and 1.3-fold (*p* = 0.004) in SKBR3 (Fig. 2g, h). Loss of expression of PDIA6 decreased survival by 71.5% (*p* < 0.0001) in HCC70 and by 42.2% (*p* < 0.0001) in SKBR3, and increased apoptosis by 2.6-fold (*p* < 0.0001) in HCC70 (Fig. 2i, j).

**Table 1:** Homology and function of selected antigens.

Name	Function	Mouse homology (%)
VPS35	Organelle trafficking	99
ARPC2	Cytoskeletal branching and cellular migration	99
SERBP1	Regulation of plasminogen activator inhibitor (PAI1).	93
KRT8	Cytoskeletal intermediate filament	95
PDIA6	Inhibition of cell death	88

### Most MHC Class II predicted epitopes derived from the human antigens inhibited tumor growth in both mouse mammary tumor models and induced a Th1 immune response in the TgMMTV-neu mouse

**Fig. 3:**
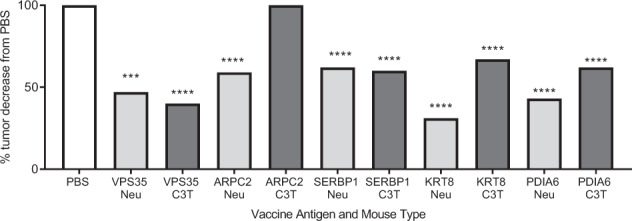
Vaccination with peptides from candidate antigen’s predicted human MHC class II epitopes inhibit tumor growth in transgenic mouse mammary tumor models. Percent decreased tumor growth from TgMMTV-neu (light gray) and C3(1)Tag mice (dark gray) *n* = 8/group as compared to control PBS vaccinated mice (white). Mice were vaccinated with peptides from VPS35, ARPC2, SERBP1, KRT8, and PDIA6 or PBS with CFA/IFA adjuvant ~14 days for three times and then with 3 × 10^5^ syngeneic mouse mammary tumor cells (MMC for TgMMTV-neu and M6 for C3(1)Tag) implanted. ****p < 0.0001.

All five targets were overexpressed in TgMMTV-neu and C3(1)Tag tumors as compared to normal mouse mammary tissue by quantitative RT PCR (Supplementary Fig. 3). Vaccination against VPS35 in TgMMTV-neu mice with implanted MMC tumors inhibited tumor growth by 47% (*p* = 0.0008, from 584.8 ± 121.8 mm^3^ to 310 ± 101.2 mm^3^), vaccination with ARPC2 inhibited tumor growth by 59% (*p* < 0.0001, from 372.7 ± 32.1 mm^3^ to 151.5 ± 17.0 mm^3^), vaccination with SERBP1 inhibited tumor growth by 62% (*p* < 0.0001, from 372.7 ± 32.1 mm^3^ to 143.7 ± 9.4 mm^3^), vaccination with KRT8 inhibited tumor growth by 31% (*p* < 0.0001, from 963.6 ± 56.7 mm^3^ to 667.3 ± 58.0 mm^3^), and vaccination with PDIA6 inhibited tumor growth by 43% (*p* < 0.0001, from 963.6 ± 56.7 mm^3^ to 553.7 ± 40.5 mm^3^) as compared to PBS controls (Fig. 3 and Supplementary Fig. 4).

Vaccination with four of the five antigens inhibited tumor growth in the C3(1)Tag mouse implanted with the M6 tumor. ARPC2 did not inhibit tumor growth in the C3(1)Tag mice and was recovered in the TgMMTV-neu screen. VPS35 vaccination inhibited tumor growth by 40% (*p* = 0.0008, from 269.5 ± 30.9 mm^3^ to 157.4 ± 17.8 mm^3^), vaccination with SERBP1 inhibited tumor growth by 60% (*p* < 0.0001, from 430.8 ± 42.8 mm^3^ to 175.8 ± 8.9 mm^3^), vaccination with KRT8 inhibited tumor growth by 67% (*p* < 0.0001, from 520.3 ± 44.5 mm^3^ to 173.8 ± 16.3 mm^3^), and vaccination with PDIA6 inhibited tumor growth by 62% (*p* < 0.0001, from 520.3 ± 44.5 mm^3^ to 198.4 ± 22.3 mm^3^) as compared to PBS controls (Fig. 3 and Supplementary Fig. 4). All tumor volumes reported were from sacrifice.

**Fig. 4:**
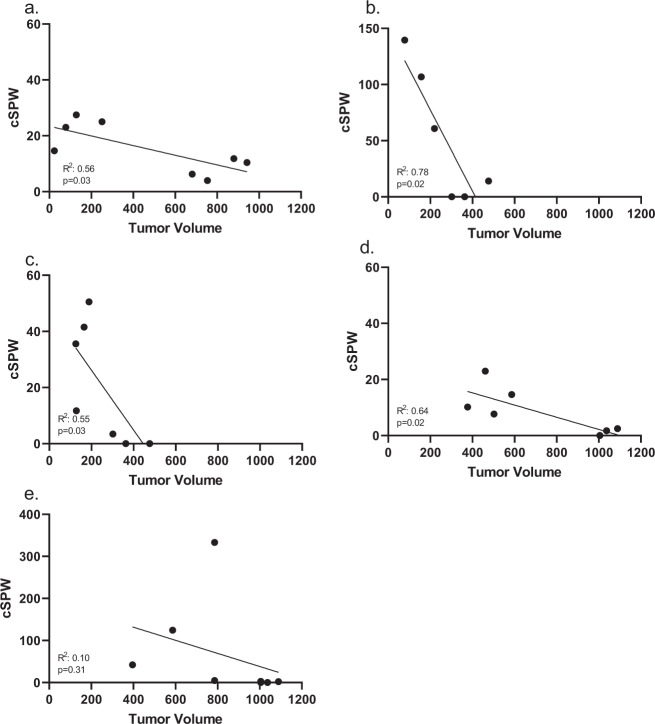
Vaccination with antigen-specific peptides was immunogenic and IFN-g immune response correlated with smaller tumor volume. Corrected spots per well (CSPW mean peptide wells as compared to no antigen wells using 3.5 × 10^5^ cells/well) was correlated by linear regression with tumor volume (mm^3^) for animals that received vaccination with pooled epitopes to a specific antigens and animals that received PBS controls. IFN-g immune response was evaluated by IFN-g ELISPOT. **a** VPS35, **b** ARPC2, **c** SERBP1, **d** KRT8, and **e** PDIA6. *R*^2^ and *p* value shown in each panel.

Occurrence of an antigen-specific IFN-g immune response was associated with smaller tumor volume in antigen vaccinated mice, as compared to PBS control mice except in PDIA6 in TgMMTV-neu mice. Mice vaccinated with pooled antigens for each of the individual targets had significantly higher average antigen-specific IFN-g ELISPOT responses than PBS control mice (Supplementary Fig. 5). An association between antigen-specific IFN-g response and smaller tumors at sacrifice were found, combining the mice vaccinated with antigen-specific peptides and mice vaccinated with PBS by linear regression analysis, except with PDIA6. This may have been because only one PDIA6 antigen induced significantly increased IFN-g T cell responses (Supplementary Fig. 5). For VPS35 the correlation between corrected spots per well VPS35-specific IFN-g T cells and tumor size was *R*^2^ was 0.56 (*p* = 0.03, Fig. 4a), for APRC2 the *R*^2^ was 0.78 (*p* = 0.02, Fig. 4b), for SERBP1 the *R*^2^ was 0.55 (*p* = 0.03, Fig. 4c), and for KRT8 the *R*^2^ was 0.64 (*p* = 0.02, Fig. 4d). The only antigen that did not have a significant correlation between the antigen-specific IFN-g T cells and tumor volume was PDIA6 with an *R*^2^ of 0.10 (*p* = 0.31) (Fig. 4e).

### Antigens elicit an immune response in early stage breast cancer

**Fig. 5:**
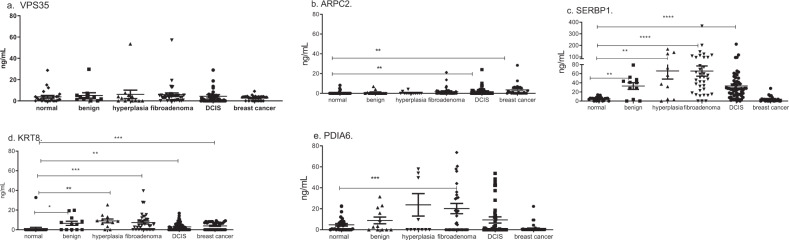
Autoantibodies against the antigens are increased in patients with breast atypia as compared to patients without breast atypia. Indirect ELISA of sera from women without breast atypia (*n* = 43) was compared to sera from women undergoing breast surgery for mammographic abnormalities (benign pathology *n* = 12, hyperplasia *n* = 12, fibroadenoma *n* = 36, or DCIS *n* = 59), and women with known breast cancer (*n* = 37). *Y* axis is ng/mL IgG antibody concentration by ELISA for each antigen and *X* axis is the breast pathology **a** VPS35, **b** ARPC2, **c** SERBP1, **d** KRT8, and **e** PDIA6 Error bars are standard error of mean (SEM) ****p < 0.0001, ***p < 0.001, **p < 0.01, *p < 0.05.

The antigens were selected based on relevance to survival of human breast cancer cell lines with HR negative HER2 positive and triple negative subtypes. Autoantibodies to the five antigens occurred in both DCIS, invasive breast cancer, and high-risk breast lesions that are benign but predict increased risk of developing breast cancer—fibroadenoma and hyperplasia^12,13^. Benign tumors were defined as radial scar, cyst, and no fibroadenoma or hyperplasia identified in women that had a mass detected on mammogram. The patients were woman and of comparable age. Only a minority of the DCIS and IBC patients had known hormone and/or HER2 receptor status (Table 2). In women with fibroadenoma, an autoantibody response occurred against VPS35 (*p* = 0.007 compared to control women, Fig. 5a), SERBP1 (*p* < 0.0001 compared to control women, Fig. 5c), KRT8 (*p* = 0.03 compared to control women, Fig. 5d), and PDIA6 (*p* = 0.003 compared to control women, Fig. 5e). When evaluating all five autoantibodies, they could distinguish women with fibroadenoma from controls with AUC 0.95 (95% CI 0.90–1.0, *p* < 0.0001) but not significantly from women with benign breast disease (Table 3). In women with hyperplasia, there were increased autoantibodies to SERBP1 (*p* < 0.0001 for controls, Fig. 5c) and KRT8 (*p* = 0.0054 for controls, Fig. 5d). The panel of autoantibodies did not distinguish women with hyperplasia from controls (Table 3). In DCIS, there were increased autoantibodies to ARPC2 (*p* = 0.03 for controls, Fig. 5b) and SERBP1 (*p* < 0.0001 for controls, Fig. 5c). Autoantibodies to all five antigens could identify women with DCIS compared to women without breast masses with AUC 0.93 (95% CI 0.88–0.98, *p* < 0.0001), and women with DCIS could be distinguished from women with benign breast masses with AUC 0.73 (95% CI 0.55–0.90, *p* = 0.01) (Table 3). There were increased autoantibodies in invasive breast cancer to ARPC2 (*p* < 0.0001 for controls, Fig. 5b) and KRT8 (*p* = 0.0004 for controls, Fig. 5d). With all five antigens, women with invasive breast cancer could be distinguished from both control women (AUC 0.80 95% CI 0.69–0.91, *p* < 0.0001) and women with benign breast masses (AUC 0.99 95% CI 0.96–1.0, *p* < 0.0001) (Table 3).

**Table 2:** Clinical and pathologic data of the serum samples.

Pathology (number)	Mean age years (range)	Hormone receptor status	HER2 receptor status	Stage
Control (43)	42 (18–74)			
Benign (12)	56 (18–78)			
Fibroadenoma (36)	54 (25–78)			
Hyperplasia (12)	56 (55–78)			
DCIS (59)	56 (34–82)	13 HR+		
		1 HR−		
		45 UNK		
IBC (37)	53 (30–74)	6 HR+ (1 HR+ HER2−)	2 HR-HER2+ 5 HR+ HER2+ 4 UNK HR HER2+ 4 HR+ HER2−	Stage I 10
		5 HR− (3 HR− HER2−)		Stage II 14
		26 UNK	22 UNK	Stage III 5
				Stage IV 8

*UNK* unknown, HR+ (ER+ PR+/ER+ PR−/ER− PR+), HER2+ (IHC 3+ or 2+ with FISH amp of HER2-neu), staging per AJCC 7. All serum samples were taken from women.

**Table 3:** Autoantibodies against the antigens detected women with breast tumors as compared to control women or women with benign tumors.

Test variable(s)	AUC	Significance	95% Confidence interval	
			Lower	Upper
DCIS vs. control	0.93	*p* < 0.0001	0.88	0.98
DCIS vs. benign	0.73	*p* = 0.01	0.55	0.90
IBC vs. control	0.80	*p* < 0.0001	0.69	0.91
IBC vs. benign	0.99	*p* < 0.0001	0.96	1.0
Hyperplasia vs. control	0.52	*p* = 0.84	0.29	0.75
Hyperplasia vs. benign	0.69	*p* = 0.13	0.47	0.91
Fibrocystic vs. control	0.96	*p* < 0.0001	0.90	1.0
Fibrocystic vs. benign	0.68	*p* = 0.07	0.50	0.86

*DCIS* ductal carcinoma in situ, *IBC* invasive breast cancer. Benign: radial scar, cyst, or no fibroadenoma or atypia found in women with mass detected by mammography.

## Discussion

We have demonstrated that antigens, identified from SEREX and natural protein array serologic studies of two transgenic mouse mammary tumor models, are recognized as immunologic targets early in tumor development and have a role for tumor survival in both mouse models. Five of these antigens (VPS35, ARPC2, SERBP1, KRT8, and PDIA6) were selected because they inhibited human breast cancer survival across two aggressive breast cancer subtype cell lines (HER2 positive and triple negative). Vaccination with most of these antigens inhibited tumor growth in both mouse models. Similar to the findings in the mouse models, autoantibodies to these antigens were increased in the serum of both DCIS and invasive breast cancer patients as compared to controls. These antigens were also increased in the serum of women with high-risk breast lesions that are non-obligate precursors of breast cancer, such as fibroadenoma and hyperplasia. These data support the use of mouse mammary tumor models to identify biologically relevant antigens with therapeutic and/or diagnostic potential in women.

The genetically engineered mouse models TgMMTV-neu and C3(1)Tag were selected because they are immunocompetent models of breast cancer, their spontaneous tumors have similar immune environments to specific human breast cancer subtypes, and they are genetically similar to luminal B (TgMMTV-neu) and triple negative (C3(1)Tag) human breast cancer^7,8^. The five antigens identified in the mouse decreased survival of triple negative and HER2 positive human breast cancer cell lines, when expression of the gene was decreased with siRNA knockdown. Vaccination with all but ARPC2 inhibited tumor growth in both mouse models. This would suggest these proteins, identified in the mouse, are potential immunogenic drivers of breast cancer that are conserved between mouse and human. In tumor development, more immunogenic cancer cells that are susceptible to T cell attack are eliminated as the tumor progresses, thus cancer initiation antigen targets may be lost if examining established invasive tumors^14^. Identification of proteins that are conserved between mouse and human may provide early targets for immunotherapeutics and diagnostics, that currently cannot be identified in breast cancer patients. The five antigens have established roles in human tumor progression. ARPC2 (actin-related protein 2/3 complex subunit 2) is an actin-related protein required for actin polymerization. ARPC2 promotes breast cancer proliferation and metastasis and is believed to be important for cell migration^15^. VPS35 (vacuolar protein sorting gene 35) manages transport from the endosomes to the Golgi. Mutations in VPS35 have been found to be associated with increased microsatellite instability in colon and hepatocellular cancer^16,17^. SERBP1 (SERPINE 1 mRNA binding protein) regulates the stability of PAI1 (plasminogen activator inhibitor 1) and is overexpressed in breast cancer^18^. SERBP1 is associated with decreased apoptosis due to altered Notch3 signaling and progression of disease in ovarian cancer^19^. KRT8 is a member of the type II keratin family and maintains cellular structural integrity as well as has roles in signal transduction and cellular differentiation. KRT8 is overexpressed in breast cancer and involved in epithelial to mesenchymal transition and cancer metastasis^20,21^. PDIA6 is protein disulfide isomerase family A member 6 and is involved in chaperoning protein folding. PDIA6 overexpression promotes metastases in breast cancer^22^. Vaccination with none of these antigens caused the mice die early. Further specific toxicity evaluation is planned with these antigens.

The five antigens important for tumor survival in the HER2 positive and triple negative human breast cancer cells lines were also important for tumor growth in vivo in both mouse models. One of the potential roles for breast cancer vaccines would be to reduce the mortality of breast cancer by preventing recurrence in women, with early breast cancer by using the immune system to destroy recurrent developing tumors^23^. Recurrent tumors can change subtypes therefore identifying antigens that have a role in tumor survival with two of the aggressive breast cancer subtypes was important^24^. Most of the known antigens identified from human studies are from advanced cancers that have developed in immunocompetent patients^25^. We have previously demonstrated that antigens identified early in mouse tumor development were more effective at inhibiting tumor growth than antigens identified in mice with established tumors in TgMMTV-neu mice. Those early antigens were neither not selected based on a role in human breast tumor survival, nor demonstrated to be essential for tumor survival in C3(1)Tag mice^6^. Four of the antigens (VPS35, SERBP1, KRT8, and PDIA6) decreased growth of the tumors in both mouse mammary tumor models (Fig. 3). This suggests these antigens have conserved roles in tumor development in mouse and human, have a role in tumor growth across aggressive human breast cancer subtypes, and may be good targets for breast cancer vaccines.

Autoantibodies have been shown to be detected prior to identification of invasive tumors by imaging and have been evaluated as biomarkers for early detection including in breast, lung, and ovarian cancer^26–28^. The autoantibody immune response seen against established tumors is an adaptive immune response that recognizes the abnormal neoplastic cells, but is ineffectual at eliminating the developing cancer^29,30^. The autoantibodies detected in this study were IgG antibodies demonstrating they are the result of a cellular immune response, because humoral class switching from IgM to IgG requires cognate T cell help^31^. Tumor-associated autoantibodies can predate the cancer diagnosis by months to years^32^. In breast cancer, three autoantibodies (PDHX, STK39, and OTUD6B) identified in TgMMTV-neu mice could also predict women who were going to develop breast cancer as compared to women, who do not develop breast cancer over 150 days prior to the detection of the breast tumor with AUC 0.68 (95% CI 0.56–0.787 *p* = 0.0003)^33^. Several autoantibody panels have been identified that can detect women with invasive breast cancer from women without known breast pathology^28,34^. For example six SEREX antigens identified in serum from breast cancer patients (RAD50, PARD3, SPP1, SAP30BP, NY-BR-62, and NY-CO-58) could identify breast cancer patients from healthy donors with 70% sensitivity and 91% specificity^20^. The five autoantibodies in this study could identify 93% of the DCIS patients and 80% of the invasive breast cancer patients as compared to control women (Table 3), and these serum samples were not selected by DCIS or breast cancer subtype (Table 2). None of the previous antibody panels evaluated the presence of the autoantibodies in high-risk breast lesions that are direct, but not obligate, precursors to invasive breast cancer (such as fibroadenoma and hyperplasia)^35,36^. All but ARPC2 of the five early antigens had increased autoantibodies in fibroadenoma as compared to control serum demonstrating that these five antigens are immunogenic early in tumor development, and the five autoantibody panel could identify 95% of patients with fibroadenoma as compared to control women. The presence of increased autoantibodies to these antigens in fibroadenoma as compared to control suggests, that the abnormal expression of these proteins develop an adaptive immune response early in tumor development^37^. Further study is needed to determine if the presence of these autoantibodies in high-risk lesions may identify more aggressive disease or can better stratify women at risk for developing breast cancer, who need more aggressive therapy.

Limitations to this study include that not all potential candidate antigens may have been identified in the SEREX and natural protein array assays due to the depths of the library screened, in the case of the SEREX screen, or the number of sequences screened in the natural protein array. Furthermore, potential targets may have been missed in the siRNA high-throughput assay because of the use of human cell lines that are immortalized and may contain mutations that make them function differently from primary breast tumors or normal breast. The human autoantibody results need to be validated with an independent serum panel and current work is ongoing to do this.

Mouse mammary tumor models of breast cancer, particularly models that represent a specific breast cancer subtype, provide a unique tool for identifying early antigens currently impossible to identify in women due to diagnostic limitations. This study demonstrates antigens, identified by SEREX and natural protein arrays in two mouse mammary tumor models of breast cancer, also have roles for survival of human HER2 positive and triple negative cancer cell lines. Furthermore, five antigens, selected because loss of expression decreases survival of human HER2 positive and triple negative breast cancer, are immunogenic in women from fibroadenoma to invasive breast cancer and, as a panel, can predict women with DCIS and IBC.

Data presented here are a proof of principle that candidate targets recovered from mouse mammary tumor models are also immunogenic in human breast cancer, and play a role in the growth of human breast cancer cell lines. Using transgenic mice to discover these targets overcomes the issue of not being able to longitudinally follow women prior to image detected tumors, to identify early breast cancer antigenic targets.

## Methods

### Mouse models

TgMMTV-neu mice (strain name: FVB/N-Tg(MMTVneu)202Mul/J, strain #002376, Jackson Laboratory, Bar Harbor, ME) were purchased from the Jackson laboratory and maintained under strict inbreeding conditions^38^. Confirmation of the transgenic strain was done using PCR for ERBB2. C3(1) Tag mice (strain name: FVB-Tg-(C3-Tag) cJeg/Jeg male mice provided from Dr. Jeff Green NCI) were mated to FVB/nJ parental females (strain #001800)^11^. TgMMTV-neu mice have a non-mutated non-activated rat *neu* under the control of the mouse mammary tumor virus (MMTV) promoter, and are not estrogen driven tumors. C3(1)Tag mice target the SV40 large T antigen to the epithelium of the mammary gland. The C3(1)Tag transgenic mice were confirmed by PCR for SV40 large T antigen (forward SV40 primer 5′-CAG AGC AGA ATT GTG GAG TGG-3′ and reverse 5′-GGA CAA ACC ACA ACT AGA ATG CAG TG-3′). TgMMTV-neu mice were confirmed by PCR for *neu* (forward primer 5′-CGG AAC CCA CAT CAG GCC-3′ and reverse primer 5′-TTT CCT GCA GCA GCC TAC GC-3′)^8^. All mice were bred under pathogen free conditions. All animal care and use was done in accordance with the University of Washington Institutional Animal Care and Use Committee guidelines.

### Serological screening of cDNA expression libraries (SEREX)

SEREX antigen screens were performed using cDNA expression libraries constructed from a syngeneic tumor cell lines (MMC for TgMMTV-neu mice and M6 for C3(1)Tag mice). MMC was derived in the Disis laboratory from spontaneous TgMMTV-neu mice^39^ and M6 cells was obtained from JE Green^40^. In brief, Poly(A)^+^ RNA was isolated from the syngeneic cells using RNA4Aqueous and Poly(A) Purist kit (Ambion, Austin TX), and the cDNA expression libraries were constructed using ZAP express vector (Strategene, La Jolla CA). A total of 3 × 10^6^ recombinant clones per library were screened. Clones were plated with XL-Blue on NZY agar plates and protein expression was induced by 4 h of incubation at 37 °C with isopropyl-l-thio-β-d-galctopyranosidase (IPTG)-impregnated nitrocellulose membrane laid on the plates^41^. Pre-diagnostic serum samples were pooled from the serum of ten mice 2 weeks prior to palpable tumor for the identification of autoantibodies at 1:200 dilution, and the membranes were incubated overnight at room temperature^33^. Alkaline phosphatase-conjugated goat anti-mouse IgG antibody (diluted 1:2000) was the secondary antibody (Thermo Fisher A16069) and nitroblue tetrazolium chloride/5-bromo-4-chloro-3-idnolyl phosphate was used for color development (Promega). The positive plaques were interrogated with pooled normal serum. Positive clones that did not react with the FVB wild-type mouse serum were purified to monoclonality and converted to pBluescript phagemid by in vivo excision using XLOLR cells (Stratagene). Plasmid DNA was prepared using Miniprep kits (Qiagen), and the nucleotide sequences of the cDNA inserts were determined using an automated DNA sequencer (ABI Prism). The sequence homology was determined by BLAST analysis^41^.

### Natural protein arrays

MMC (for TgMMTV-neu) and M6 (for C3(1)Tag) syngeneic cell lines were separated by anion exchange HPLC as described previously^1,2,42^. Briefly, each of the cell lysates were subjected to orthogonal two-dimensional high-performance liquid chromatography (2D-HPLC) fractionation in an automated system (Shimadzu Corporation). An excess of protein from each cell line was fractioned to ensure adequate protein content in arrayed spots and availability of protein fractions for further investigation and validation. Fractionation was based ion anion exchange (SAX/10 column, 7.5 mm ID_150 mm, Column Technology Inc.) using a 40 step-elution, followed by a second dimension reversed-phase separation (RP/5D column, 4.6mmID_150 mm, Column Technology Inc.). The first dimension anion-exchange chromatography mobile phase A was 20 mmol/L Tris, pH 8.5, and mobile phase B was 20 mmol/L Tris, 1 mol/L NaCl, and pH 8.5. The second dimension reversed-phase chromatography mobile phase A was 95% water, 5% acetonitrile, and 0.1% trifluoroacetic acid (TFA), and mobile-phase B was 90% acetonitrile, 10% water, and 0.1% TFA.

A total 1840 protein fractions from each cell line were printed onto nitrocellulose-coated slides (Schleicher & Schuell, Keane, NH). The microarrays were air-dried and rinsed with PBST (PBS with 0.5% Tween-20) for 30 min, then blocked with TBST (TBS with 0.1% Tween-20) for 1 h. Serum samples were diluted 1:150 in PBST (PBS with 0.1% Tween-20), applied to the slides and incubated for 1.5 h. The microarrays were washed with PBST (PBS with 0.1% Tween-20) and incubated with 5 μg/mL biotinylated anti-human IgG antibody (Amersham, Piscataway, NJ) diluted in 3% nonfat milk/PBST. Following a brief wash in PBST (PBS with 0.1% Tween-20), the microarrays were incubated with 5 μg/mL R-Phycoerythrin conjugated streptavidin (Molecular Probe, Eugene, OR) diluted in 3% nonfat milk/PBST (PBS with 0.1% Tween-20) for 30 min. The microarrays were developed with 5 μg/mL biotinylated anti-streptavidin antibody diluted in 3% nonfat milk/PBST (PBS with 0.1% Tween-20) and 5 μg/mL R − Phycoerythrin-conjugated streptavidin diluted in 3% nonfat milk/PBST (PBS with 0.1% Tween-20). The microarrays were dried by spinning at 750 × *g* for 2 min.

The microarrays were scanned with a GenePix scanner using a green laser (532 nm) at constant detector gain and power setting for all slides. Scanned images were analyzed using ImaGene 5.0 image analysis software. Local background subtracted median spot intensities were used for downstream statistical analysis.

### siRNA knockdown screen

In the large scale screen, each target gene was knocked down with a pool of four unique siRNAs. Cell lines representing human triple negative (HCC70) and hormone receptor negative HER2 positive breast cancer subtype (SKBR3) were compared to a non-malignant human breast cell line (MCF10F). All cell lines were purchased from ATCC (Manassas VA) and tested for mycoplasma by the universal mycoplasma detection kit (ATCC) prior to the large scale screens. Transfection conditions, cell number, timing, and reagent concentration were determined by feasibility studies that identified optimal assay conditions to obtain a ~20-fold window using a positive apoptosis control siRNA associated with cell survival (Kif11 siRNA, Qiagen Germantown PA), and universal negative control siRNA (AllStars Negative Control siRNA, Qiagen). For confirmation of the large scale screen, the pooled siRNA was deconvoluted, manually transfected in 96-well format, and assayed for cell survival and apoptosis. The positive control was AllStars HS Cell Death Control (Qiagen). For the large scale screens, candidate antigens were chosen based on (a) ≥30% decreased viability and (b) ≥1.5-fold increased apoptosis in the invasive breast cancer cell lines, but not in the non-malignant cell line.

### Apoptosis assay

Apoptosis was measured with a caspase 3/7 fluorescence assay (Promega, Madison WI). For MCF10F 100 μL caspase 3/7 reagent was added per well and for HCC70 and SKBR3 40 μL of caspase 3/7 reagent was added per well. The plates were then incubated for 30 min at 37 °C and read by luciferase intensity using the Wallac Envision 2104 Multi-label Detector/plate reader with a 96-well aperture (Perkin Elmer, Waltham MA).

### Cell viability assay

Cell viability was measured by ATP quantification using Cell Titer Glo (Promega, Madison WI). Twenty microliter of cell titer glow reagent was added per well and read using the Wallac Envision 2104 Multi-label Detector/plate reader with a 96 well aperture (Perkin Elmer, Waltham MA).

### In vivo murine vaccine studies

Peptides were predicted from human protein sequences by a published computer algorithm to identify putative MHC class II epitopes^43^. Fifteen to twenty-five amino acid peptides were constructed to encompass ~20% of the protein sequence for each candidate (Supplementary Table 2). Peptides selected had >90% amino acid homology between mouse and human. Fifty microgram of the peptides pooled by antigen were mixed with complete Freund’s adjuvant (1st injection) or incomplete Freund’s adjuvant (subsequent injections) and given by intradermal injection. Eight mice per group were vaccinated three times 14 days apart. A syngeneic MMC mouse carcinoma line in TgMMTV-neu^39^ or M6 carcinoma line in C3(1)Tag^40^ were implanted 1 week after vaccination. The MMC cell line was verified to express rat neu (Mb 7.16.4, mouse IgG2a primary anti-rat *neu* p185 OP16L, Millipore Burlington, MA) and secondary FITC-conjugated anti-rat anti mouse (Clone A85-1 RUO catalog number 562026, BD Pharmagen San Jose CA) by flow cytometry, and the M6 cell line was verified to express the SV40 antigen by western blotting (mouse anti-SV40 large T antigen catalog number 554149, BD Pharmagen). Mice were inoculated with 3 × 10^5^ MMC^39^ or M6 cells^40^ subcutaneously on the mid-dorsum with a 23-gauge needle. Tumors were measured 3x/week with Vernier calipers and tumor volume was calculated as the product of length × width × height × *π*/6. Mice were euthanized when the tumor ulcerated, was >1000 mm^3^, or if the animal was ~1 year old.

### IFN-g ELISPOT

Splenocytes from the TgMMTV-neu mice vaccinated with the peptides from the five target antigens or PBS controls (*n* = 4 mice/group) and IFN-g ELISPOT was performed as previously described^9^. In brief, 3.5 × 10^6^ cells/mL in six replicates were stimulated with antigen-specific peptides, CONA (positive control), or HIV Gag p17 (negative control)^44^ in 96-well PVDF (MAIPS4510, Fisher) plates that had been incubated overnight with 10 μg/mL anti-mouse IFN-g (clone:AN18, MabTech, Cincinnati OH). After 72 h, the plates were incubated with biotinylated anti-mouse IFN-g (Clone: R4-6A2-biotin, MabTech, Cincinnati OH), and developed with streptavidin-alkaline phosphatase. The spots were quantified using an AID ELISPOT High-Resolution reader system (software version 6.0). Data are reported as corrected spots per well (CSPW), the mean spots for each experimental antigen subtracted from the mean number of spots from no antigen wells.

### ELISA

ELISAs were performed as described previously^33^. In brief, serum from control women (*n* = 43) were acquired from volunteers from the Puget Sound Blood Center and serum from women with invasive breast cancer (*n* = 37) were acquired from the Cancer Vaccine Institute specimen repository and were collected using the same methods (Table 2). The serum for benign, fibroadenoma, hyperplasia, and DCIS were obtained from a Duke repository with blood drawn at the time of an abnormal mammogram, and in patients with no invasive pathology at surgery. The definition of a benign lesion was defined as radial scar, cyst, and no fibroadenoma or hyperplasia. The Duke Repository serum was from women with benign tumors (*n* = 12), fibroadenoma (*n* = 36), hyperplasia (*n* = 12), and DCIS (*n* = 59) (Table 2). All patients were consented prior to blood collection at the institution where the samples were obtained however, because all the samples were de-identified before the investigators for this study received them, they were IRB exempt as determined by the University of Washington institutional review board. Each serum sample was evaluated individually by indirect ELISA with commercially available human recombinant proteins. In brief, full length human recombinant proteins were coated onto 96-well Immulon 2B plates (Fisher) overnight alternating with a carbonate buffer blank. Human recombinant proteins were obtained from Origene (ARPC2 TP300319, SERBP1 TP301092, PDIA6 TP301710, and KRT8 TP309570) or Novus (VPS35 H00055737-P01). Positive controls were VPS35 goat polyclonal (Abnova ab10099 1:5000), ARPC2 goat polyclonal (Abnova PAB6168 1:1000), SERBP1 mouse monoclonal (Abnova H00026135, 1:25,000), KRT8 mouse monoclonal (Abnova MAB9946 1:25,000), and PDIA6 mouse monoclonal (H00010130-M04 Abnova 1:200). Experimental serum samples were evaluated in 1:200 dilution with horseradish peroxidase conjugated goat anti-human IgG (Thermo Fisher goat anti-human catalog # 65-6120, RRID AB_2533967), rabbit anti-goat IgG (Thermo Fisher Scientific, catalog # 31460, RRID AB_228341), or goat anti-mouse IgG (Thermo Fisher Scientific, catalog # 62-6520, RRID AB_2533947) diluted to 1:5000 as secondary antibody. The plates were developed with TMB peroxidase (SeraCare, Milford MA). After development, the plates were read at absorbance of 450 nm. The OD of each serum was calculated as the OD of the antigen-coated wells minus the OD of the non-antigen coated carbonate buffer wells. The autoantibody concentration (ng/well) was calculated from a 4-parameter fitted standard curve on each plate. Positive controls were a polyclonal antibody developed against the full length human target protein. All samples were evaluated in quadruplicate. All ELISAs were confirmed by western blot with >75% specificity and >75% sensitivity between the two assays (e.g., Supplementary Fig. 2).

### Statistical analysis

For the volcano plot, the *p* values of the caspase/viability ratio of either HCC70 or SKBR3 was compared to the caspase/viability ratio of MCF10F by a student *t*-test. The – log(10) of the *p* value of the difference between cancer and non-malignant cell lines, and the log (2) of the cancer cell caspase/viability ratio to the non-malignant caspase/viability was calculated in Microsoft Excel. The –log(10) of the *p* value was graphed against log (2) of the cancer cell caspase/viability ratio to the non-malignant caspase/viability ratio in Graph Pad Prism v6.05 software. A two-way ANOVA with Bonferroni’s post-test correction was used for comparisons between mouse groups for tumor growth. A one-way ANOVA with Dunnett’s comparison was used in rtPCR siRNA knockdown analyses, cell survival/apoptosis analyses, and evaluation of ELISA data. The sensitivity and specificity of the combination of antibodies in human serum samples was evaluated using receiver-operating-characteristic analysis based on a linear regression model with maximum likelihood estimation. Area under the curve with 95% confidence intervals were calculated using SPSS software, version 25. Significance was considered at *p* < 0.05 for all statistical tests except in the large scale screens, where significances was considered at *p* < 0.1.

### Reporting summary

Further information on research design is available in the Nature Research Reporting Summary linked to this article.

## Supplementary information

Reporting Summary

Supplementary Data

## Data Availability

The data generated and analysed during this study are described in the following data record: 10.6084/m9.figshare.14333726^45^. The data are contained in Excel spreadsheets in the files “NPJ to make volcano.xlsx” and “NPJ Figures.xlsx”. These files are not openly available in a repository as they contain additional data that were not included in the related study. However, the files are available upon request by email to the corresponding author.

## Code Availablity

N/A.
