# Supplementary material for: Tumor-associated autoantibodies from mouse breast cancer models are found in serum of breast cancer patients
Source: NPJ Breast Cancer. 2021 May 11;7:50. doi: 10.1038/s41523-021-00257-1 (PMC8113561; doi:10.1038/s41523-021-00257-1)
Supplement: Supplementary file 1 — Reporting Summary [file 41523_2021_257_MOESM1_ESM.pdf]

nature research

Corresponding author(s): Sasha Stanton

Last updated by author(s): Jan 25, 2021

Reporting Summary

Nature Research wishes to improve the reproducibility of the work that we publish. This form provides structure for consistency and transparency in reporting. For further information on Nature Research policies, see our Editorial Policies and the Editorial Policy Checklist.

Statistics

For all statistical analyses, confirm that the following items are present in the figure legend, table legend, main text, or Methods section.

n/a	Confirmed	
☐	☒	The exact sample size (n) for each experimental group/condition, given as a discrete number and unit of measurement
☐	☒	A statement on whether measurements were taken from distinct samples or whether the same sample was measured repeatedly
☐	☒	The statistical test(s) used AND whether they are one- or two-sided<br>*Only common tests should be described solely by name; describe more complex techniques in the Methods section.*
☒	☐	A description of all covariates tested
☐	☒	A description of any assumptions or corrections, such as tests of normality and adjustment for multiple comparisons
☐	☒	A full description of the statistical parameters including central tendency (e.g. means) or other basic estimates (e.g. regression coefficient) AND variation (e.g. standard deviation) or associated estimates of uncertainty (e.g. confidence intervals)
☐	☒	For null hypothesis testing, the test statistic (e.g. F, t, r) with confidence intervals, effect sizes, degrees of freedom and P value noted<br>*Give P values as exact values whenever suitable.*
☒	☐	For Bayesian analysis, information on the choice of priors and Markov chain Monte Carlo settings
☒	☐	For hierarchical and complex designs, identification of the appropriate level for tests and full reporting of outcomes
☐	☒	Estimates of effect sizes (e.g. Cohen's d, Pearson's r), indicating how they were calculated

Our web collection on statistics for biologists contains articles on many of the points above.

Software and code

Policy information about availability of computer code

Data collection	No code was developed or used
Data analysis	The analysis was performed using Microsoft Excel 2011, GraphPad Prism v6.05, and SPSS version 25.

For manuscripts utilizing custom algorithms or software that are central to the research but not yet described in published literature, software must be made available to editors and reviewers. We strongly encourage code deposition in a community repository (e.g. GitHub). See the Nature Research guidelines for submitting code & software for further information.

Data

Policy information about availability of data

All manuscripts must include a data availability statement. This statement should provide the following information, where applicable:
- Accession codes, unique identifiers, or web links for publicly available datasets
- A list of figures that have associated raw data
- A description of any restrictions on data availability

The datasets generated during and/or analysed during the current study are available from the corresponding author on reasonable request.

Field-specific reporting

Please select the one below that is the best fit for your research. If you are not sure, read the appropriate sections before making your selection.

☒ Life sciences ☐ Behavioural & social sciences ☐ Ecological, evolutionary & environmental sciences

For a reference copy of the document with all sections, see nature.com/documents/nr-reporting-summary-flat.pdf

Life sciences study design

All studies must disclose on these points even when the disclosure is negative.

Sample size	For the mouse studies comparing adjuvant immunized mice to antigen vaccinated, eight animals per group was used to provide 95% power to observe a statistically significant difference in tumor size between antigen vaccinated mice as compared to control vaccinated mice (at the two-sided level of α=0.05) if the effect size is 2.0. For the human samples, screening 30 DCIS cases with 30 matched controls gives a 90% power to detect a 2-fold difference in candidate antigen specific IgG (µg/ml) between control and DCIS patients as calculated by the observed distribution of IgG response in a previous antigen (IGF-IR) in breast cancer (Cecil Breast Cancer Res Treat 139:657)
Data exclusions	No data were excluded.
Replication	The large scale siRNA screen was repeated in its entirety twice. The small scale was performed in triplicate. All the RT PCR assays were performed in quadruplicate. All IFN-g ELISPOT assays were performed with 6 replicates. The mouse studies were not performed twice as the TgMMTV-neu and C3(1)Tag studies were used as replicates for eachother.
Randomization	No randomization was performed, not appropriate for this study
Blinding	No blinding was performed for this study but the validation serum set IS blinded.

Reporting for specific materials, systems and methods

We require information from authors about some types of materials, experimental systems and methods used in many studies. Here, indicate whether each material, system or method listed is relevant to your study. If you are not sure if a list item applies to your research, read the appropriate section before selecting a response.

Materials & experimental systems

n/a	Involved in the study	
☐	☒	Antibodies
☐	☒	Eukaryotic cell lines
☒	☐	Palaeontology and archaeology
☐	☒	Animals and other organisms
☐	☒	Human research participants
☒	☐	Clinical data
☒	☐	Dual use research of concern

Methods

n/a	Involved in the study	
☒	☐	ChIP-seq
☒	☐	Flow cytometry
☒	☐	MRI-based neuroimaging

Antibodies

Antibodies used	Antibodies for positive controls used for ELISAs: ARPC2 goat polyclonal PAB618 (Abnova), VPS35 goat polyclonal AB10099 (Abnova), SERBP2 mouse monoclonal H00026135 (Abnova), KRT8 mouse monoclonal MAB9946 (Abnova), and PDIA6 mouse monoclonal H00010130-M04 (Abnova). Secondary antibodies IgG rabbit anti-goat HRP (AB_2535915, ThermoFisher) and IgG goat anti-mouse HRP (62-6520 ThermoFisher) . For IFN-g ELISPOT, Primary AN18 (MabTech) and secondary R4-6A2-biotin (MabTech)
Validation	For mouse ELISPOT the antibodies were validated by MABTECH. All human antibodies and recombinant proteins were first tested by checkerboard analysis to determine optimal concentration of secondary antibodies and recombinant proteins. Furthermore, all human antibodies were validated by Western Blot (example see Supplemental Figure 2)

Eukaryotic cell lines

Policy information about cell lines

Cell line source(s)	ATCC and laboratory developed mouse cell lines.
Authentication	Cells that were directly obtained directly from ATCC were not authenticated because they were purchased directly from the repository. The MMC cell line was verified to express rat neu by flow cytometry and the M6 cell line was verified to express the SV40 antigen by western blotting, and to be estrogen receptor negative by rtPCR.

Mycoplasma contamination	All cell lines were tested for mycoplasma by the universal mycoplasma detection kit (ATCC) prior to the large scale screens or implant into the mouse.
Commonly misidentified lines (See ICLAC register)	none

Animals and other organisms

Policy information about studies involving animals; ARRIVE guidelines recommended for reporting animal research

Laboratory animals	All mice were female as in these models they develop breast cancer. TgMMTV-neu mice (strain name: FVB/N-Tg(MMTVneu)202Mul/J, strain #002376, Jackson Laboratory, Bar Harbor, ME) were purchased from the Jackson laboratory and maintained under strict inbreeding conditions.(38) Confirmation of the transgenic strain was done using PCR for ERBB2. C3(1) Tag mice (strain name: FVB-Tg-(C3-Tag) cJeg/Jeg male mice provided from Dr. Jeff Green NCI) were mated to FVB/nJ parental females (strain #001800). The C3(1)Tag transgenic mice were confirmed by PCR for SV40 large T antigen.
Wild animals	none
Field-collected samples	none
Ethics oversight	All animal care and use was done in accordance with the University of Washington Institutional Animal Care and Use Committee guidelines.

Note that full information on the approval of the study protocol must also be provided in the manuscript.

Human research participants

Policy information about studies involving human research participants

Population characteristics	The serum from control women (n=43) were acquired from volunteers from the Puget Sound Blood Center and serum from women with invasive breast cancer (n=37) were acquired from the Cancer Vaccine Institute specimen repository and were collected using the same methods (table 3). The serum for benign, fibroadenoma, hyperplasia, and DCIS were obtained from a Duke repository with blood drawn at the time of an abnormal mammogram and in patients with no invasive pathology at surgery. The definition of a benign lesion was defined as radial scar, cyst, and no fibroadenoma or atypia. The Duke Repository serum was from women with benign tumors (n=12), fibroadenoma (n=37), hyperplasia (n=12), and DCIS (n=59)
Recruitment	These samples were from repositories therefore the recruitment is not known.
Ethics oversight	These samples had no clinically identifiable data associated with them therefore it did not need IRB approval. Our clinical research coordinator (Jennifer Childs) confirmed that the samples did not need IRB approval with the University of Washington IRB.

Note that full information on the approval of the study protocol must also be provided in the manuscript.
