# Supplementary material for: Tumor-associated autoantibodies from mouse breast cancer models are found in serum of breast cancer patients
Source: NPJ Breast Cancer. 2021 May 11;7:50. doi: 10.1038/s41523-021-00257-1 (PMC8113561; doi:10.1038/s41523-021-00257-1)
Supplement: Supplementary file 2 — Supplementary Data [file 41523_2021_257_MOESM2_ESM.pdf]

Supplementary Files

Supplementary Methods:

siRNA IDs for the selected antigens:

All siRNA were ordered from Qiagen. VPS35 the siRNA used were SI04316914, SI04287605, SI04279296, and SI04268131. For APRC2 the siRNA used were SI00299264, SI04297916, SI04216884, and SI04208218. For SERBP1 the siRNA used were SI04348372, SI04265044, SI04256182, and SI04249350. For KRT8 the siRNA used were SI03083304, SI03058356, SI03055248, and SI03035214. For PDIA6 the siRNA used were SI03097871, SI03097871, SI00753690, and SI00753683.

siRNA IDs for all antigens:

All siRNA were ordered from Qiagen. For ALDOA the siRNA used were SI03118045, SI03071005, SI03070340, and SI03069675. For ALDOC the siRNA used were SI04294101, SI04201897, SI04157944, and SI00294308. For APRC2 the siRNA used were SI00299264, SI04297916, SI04216884, and SI04208218. For DNAJB14 the siRNA used were SI04334561, SI04206076, SI03224935, and SI03172792. For ENO1 the siRNA used were SI02654309, SI02653798, SI03065755, and SI03054905. For HIST1H1B the siRNA used were SI04371983, SI00436702, SI00436695, and SI00436688. For HIST1H1D the siRNA used were SI04209513, SI04175444, SI04158567, and SI04135376. For HIST1H2BA the siRNA used were SI04359320, SI04226509, SI04187372, and SI00437185. For HIST1H2BC the siRNA used were SI04370471, SI04139352, SI03230360, and SI03147900. For HIST1H2BD the siRNA used were SI04949161, SI03190712, SI03132381, and SI00437248. For HIST1H2BH the siRNA used were SI03211950, SI00437381, SI00437367, and SI00437360. For HNRPA2B1 the siRNA used were SI02663486, SI02663479, SI00300426, and SI03029229. For KRT18 the siRNA used were SI02653658, SI04313540, SI03234091, and SI03187996. For KRT7 the siRNA used were SI04339776, SI04294521, SI00465066, and SI00465052. For KRT8 the siRNA used were SI03083304,

SI03058356, SI03055248, and SI03035214. For MDH2 the siRNA used were SI00082698, SI00082691, SI00082684, and SI00082677. For NME1 the siRNA used were SI02665341, SI02663584, SI02663577, and SI00287343. For NME2 the siRNA used were SI05151804, SI05151790, SI04777528, and SI04771613. For NSFL1C the siRNA used were SI04336248, SI04333721, SI03150728, and SI00661815. For PDIA3 the siRNA used were SI02654778, SI02654771, SI03075485, and SI03025442. For RBM3 the siRNA used were SI04297755, SI04296166, SI04261215, and SI04146821. For RPS7 the siRNA used were SI04336416, SI04280360, SI04280164, and SI03191006. For SERBP1 the siRNA used were SI04348372, SI04265044, SI04256182, and SI04249350. For SFRS3 SI04315276, SI04311531, SI04264771, and SI04207756. For SFRS7 the siRNA used were SI04267529, SI04157216, SI04146401, and SI00716499. For ZNF518 the siRNA used were SI03177461, SI04131015, SI04284805, and SI04336129. For PDHX the siRNA used were SI05348581, SI05348574, SI04343682, and SI04298476. For STK39 the siRNA used were SI02224404, SI02224397, SI04439365, and SI04439358. For OTUD6B the siRNA used were SI04233915, SI04185034, SI04172420, and SI00344939. For LGALS8 the siRNA used were SI04356506, SI04340028, SI04336612, and SI03237339. For DNAJC10 the siRNA used were SI02777852, SI03024133, SI00371014, and SI00371007. For VPS35 the siRNA used were SI04316914, SI04287605, SI04279296, and SI04268131. For ADAMTS2 the siRNA used were SI03105368, SI03103555, SI03098935, and SI03068310. For DKC1 the siRNA used were SI03080616, SI03040744, SI00364602, and SI00364581. For EIF5A the siRNA used were SI00300132, SI04300380, SI04283027, and SI04236911. For HSPA5 the siRNA used were SI02781016, SI02780554, SI03090682, and SI00075943. For HSPA9 the siRNA used were SI02654813, SI04355043, SI04319189, and SI04279884. For IDH3A the siRNA used were SI00300524, SI04256644, SI04229407, and SI04209723. For KRT14 the siRNA used were SI04165532, SI00464429, SI00464422, and SI00464415. For KRT19 the siRNA used were SI04158238, SI03184944, SI03156356, and SI00464527. For KRT4 the siRNA used were SI04371549, SI04330431, SI04277217, and

SI04258366. For KRT6A the siRNA used were SI04656848, SI04656841, SI04348064, and SI04250666. For KRT9 the siRNA used were SI04321366, SI04222582, SI04148221, and SI04143097. For NUP153 the siRNA used were SI03033226, SI03031294, SI00072786, and SI00072779. For OGFR the siRNA used were SI03032162, SI02638762, SI02638755, and SI00096523. For PDIA6 the siRNA used were SI03097871, SI03097871, SI00753690, and SI00753683. For PKM2 the siRNA used were SI02659930, SI00288022, SI03110583, and SI03032211. For PSMC3 the siRNA used were SI00301462, SI00043533, SI00043526, and SI00043519. For PTPBP1 the siRNA used were SI00141638, SI00043631, SI03087049, and SI02649206. For S100A11 the siRNA used were SI04145092, SI04133766, SI03166723, and SI03132878. For SFSF2 the siRNA used were SI00301777, SI05461855, SI04358109, and SI04270637. For STXBP3A the siRNA used were SI04327988, SI04321996, SI00735742, and SI00735735. For TPI1 SI04285141, SI04282628, SI04192713, and SI03137379. For U2AF2 the siRNA used were SI04194498, SI04146135, SI00754026, and SI04290153. For UBB the siRNA used were SI03115105, SI03107328, SI03059280, and SI00754243. For UBC the siRNA used were SI03048423, SI00754285, SI00754278, and SI00754271. For VIM the siRNA used were SI02655198, SI00302197, SI00302190, and SI04201890. For WHSC1 the siRNA used were SI04951275, SI03029936, SI02622704, and SI02622697. For ZFP238 the siRNA used were SI04348281, SI04285785, SI04220181, and SI00770623. For SHOX2 the siRNA used were SI04296411, SI04141991, SI03150329, and SI00718088. For USP1 the siRNA used were SI04362631, SI04346041, SI04302207, and SI03155439. For CANX the siRNA used were SI02757300, SI02663367, SI04434444, and SI00027650. For UBFD1 the siRNA used were SI02778251, SI02778258, SI00124145, and SI03027563.

Antigen-specific silencing with siRNA. Each siRNA was transfected individually into 150,000 cells MCF10F in six-well plates. Forty-eight hours after transfection the cells were collected by

scraping and total RNA was produced using RNA-easy kit (Ambion). The stability and purity of the RNA was confirmed by nanodrop and PCR for tRNA common proteins (forward primer reverse primer. Controls included liposomal transfected (control), negative control transfected cells, untransfected cells, water control, and a no reverse transcriptase control.

mRNA quantitation. For quantitation of expression of each target in mice, total mRNA was made from tumor or mammary tissue using RNAqueous for PCR (Ambion, Austin TX), and reverse transcribed using SuperScript III First-Strand Synthesis System (Invitrogen, Grand Island NY). Real-time PCR was done using a StepOne plus real time PCR machine (Thermofisher). All PCR reactions used the Taqman master mix and Taqman Primer/probe sets. The mouse primer/ probe sets were VPS35 (Mm00458167_m1), ARPC2 (Mm01254383_m1), SERBP1 (Mm00482136_m1), KRT8 (Mm04209403_g1), and PDIA6 (Mm01276904_m1). All Taqman primer/probe sets were purchased from Applied Biosystems (Invitrogen, Grand Island NY). All assays were run in quadruplicate. mRNA expression level was normalized to mouse b-actin using the ΔC_T method where level of expression is equal to $2^{-\Delta C_T}$, where $\Delta C_T = C_T$ antigen - C_T actin. C_T is the cycle threshold at which the fluorescence signal crosses an arbitrary value.

Supplementary Table 1: Sixty three autoantibodies identified in mice prior to palpable tumor development tumors

TgMMTV-neu	C3(1)Tag
ALDOA	ADAMTS2
ALDOC	CANX
ARPC2	DKC1
DNAJC10	EIF5A
DNAJB14	ENO1
ENO1	HSPA5
HIST1H1B	HSPA9
HIST1H1D	IDH3A
HIST1H2BA	KRT14
HIST1H2BC	KRT18
HIST1H2BD	KRT19
HIST1H2BH	KRT4
HNRNPA2B1	KRT6A
KRT18	KRT7
KRT7	KRT9
KRT8	NUP153
LGALS8	OGFR
MDH2	PDIA3
NME1	PDIA6
NME2	PKM2
NSFL1C	PSMC3
OTU6B	PTPBP1
PDIA3	S100A11
PDHX	STXBP3A
PKM2	TPI1
RBM3	U2AF2
RPS7	VIM
SERBP1	SHOX2
SRSF3	SRSF2
SRSF7	UBB
STK39	UBC
UBFD1	USP1
VPS35	WHSC1
ZFP238	
ZNF518B	

Proteins in light gray were recovered from SEREX screening, proteins in black from natural protein arrays, and proteins in dark gray were recovered in both mouse models.

Supplementary Table 2 Predicted human MHC class II peptide sequences used for vaccine studies:

	NCBI human	NCBI mouse	Human Peptide Sequence	Homology by amino acid to mouse
**VPS35** p108-126	NP_060676.2	NP_075373.1	LYLLITVGVVYVKSFPQSR	100%
p146-160			GLFLRNYLLQCTRNI	100%
p182-202			FVLLNFAEMNKLWVRMQHQGH	100%
p321-336			DIKLFDIFSQQVATVI	100%
p439-455			YVLSNVLDYNTEIVSQD	100%
p507-522			YLILNTARKHFGAGGN	100%
**ARPC2** p55-77	NP_690601.1	NP_001344316.1	VMVSISLKFYKELQAHGADELLK	100%
p249-270			DYLHYHIKCSKAYIHTRMRAKT	100%
**SERBP1** p6-25	AAH26916.1	AAH30502.1	QEGFGCVVTNRFDQLFDDES	100%
p101-118			LKKEGIRRVGRRPDQQLQ	100%
p343-364			HFRKPANDITSQLEINFDGLGR	100%
**KRT8** p31-p50	AAH08200.1	AAH94009.1	LQSQISDTSVVLSMDNSRSL	100%
p170-189			YQELMNVKLALDIEIATYRK	95%
**PDIA6** p4-25	NP_005733.1	NP_082235.2	LVLGLVSCTFFLAVNGLYSSSD	100%
p122-141			AIVDAALSALRQLVKDRLGG	100%
p354-373			FGYPAMAAINARKMKFALLK	100%

Amino acid sequence differences between mouse and human in red.

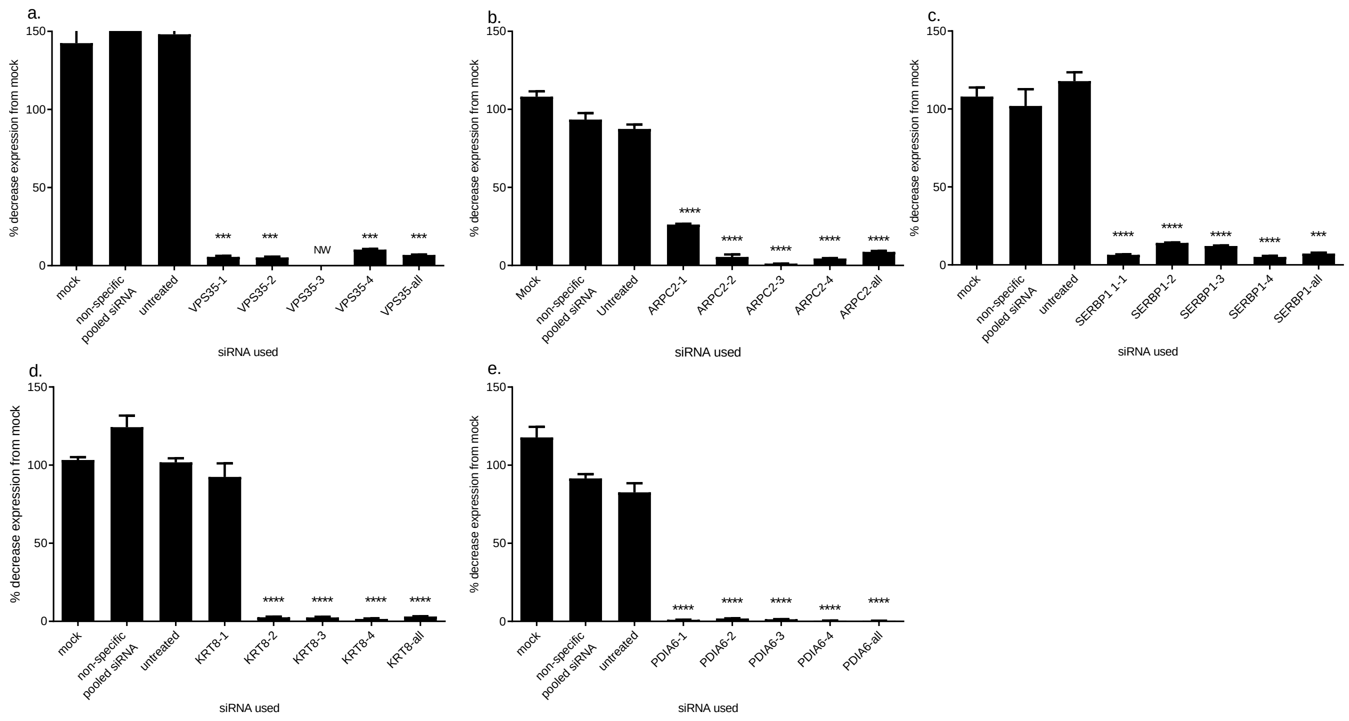

Supplementary Figure 1: Target-specific decreased expression of the four siRNA for each target by RT PCR in the MCF10F cell line. (a) VPS35 (b) ARPC2 (c) SERBP1 (d) PDIA6 and (e) KRT8 Y is % decreased expressed compared to mock and X is the siRNA transfected. All is the four siRNA pooled. Error bars are standard error of mean (SEM). *** p=0.001 **** p<0.0001 NW=assay did not work

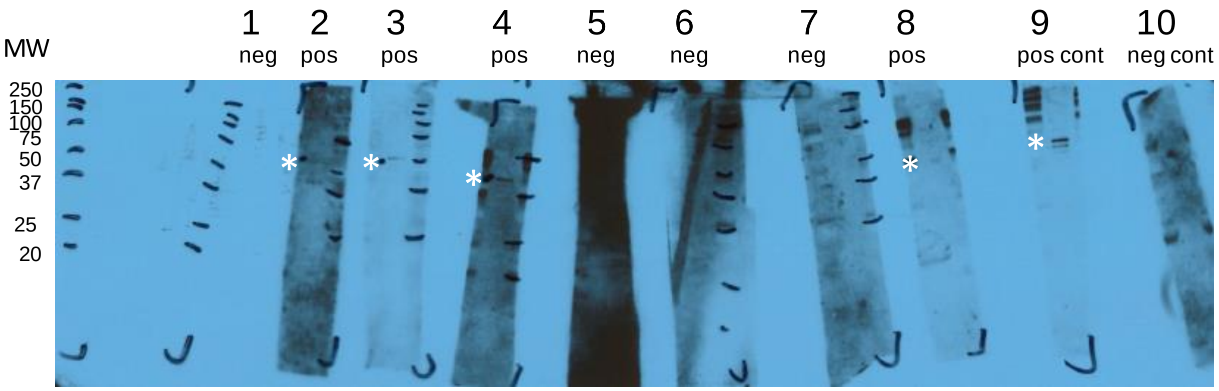

Supplementary Figure 2: Western blot confirmation of ELISA results for KRT8. Recombinant human protein was run on acrylamide gel and blotted with four serum samples that had been positive by KRT8 ELISA (pos, lanes 2,3, 4, and 8) and four serum samples that had been negative by KRT8 ELISA (neg, lanes 1, 5, 6 and 7). White stars marks KRT8 at 55 kDa (confirmed by the positive control (pos cont) human monoclonal KRT8 antibody lane 9 and negative control horse myoglobin (neg cont) lane 10). MW is the molecular weight ladder included (in kDa). 4/4 samples that were positive in ELISA were positive by Western and 4/4 samples that were negative by ELISA were negative by Western making the sensitivity 100% and specificity 100%. Gel derives from the same experiment and all samples processed in parallel.

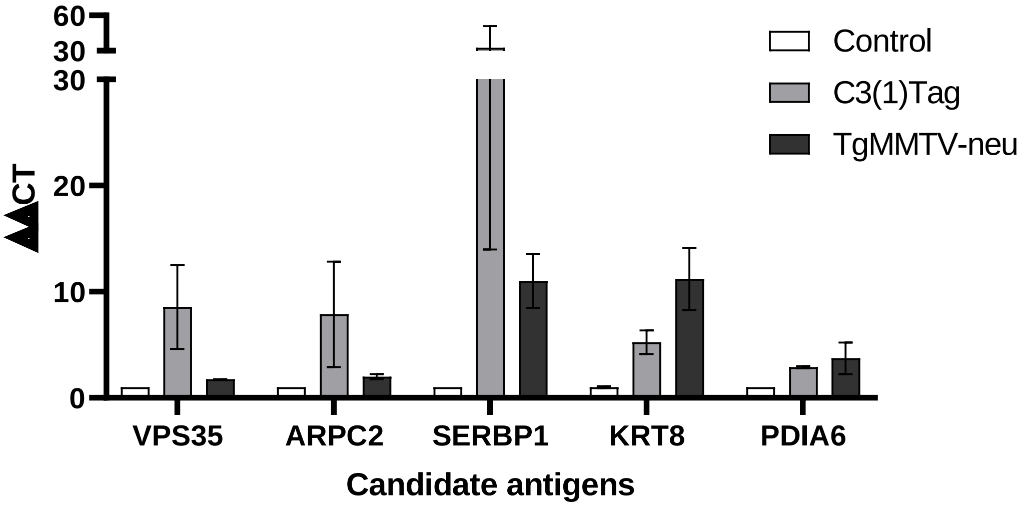

Supplementary Figure 3: mRNA for all five antigen targets are overexpressed in both TgMMTV-neu and C3(1)Tag mouse mammary tumors as compared to FVB mammary tissue. Tumors from two TgMMTV-neu, two C3(1)Tag mice, and two normal mouse breast samples had mRNA produced using RNAqueous for PCR. All four tumors were collected at 300 mm^3. RT PCR for each of the target antigens demonstrate that the mRNA for each antigen is overexpressed in both C3(1)Tag (light gray) and TgMMTV-neu (dark gray) tumors but not in normal FVB mouse breast (white) using mouse primers. Each RT PCR was performed in quadruplicate in two individual tumors. Y axis is normalized mRNA expression level to mouse b-actin using the $\Delta\Delta C_T$ method where mRNA expression is equal to $2^{-\Delta C}{}_T$ where $\Delta C_T = C_T$ antigen - C_T actin. C_T is the cycle threshold at which the fluorescence signal crosses an arbitrary value. The x axis is the candidate antigens and the error bars are differences between the two samples. Error bars are standard error of mean (SEM).

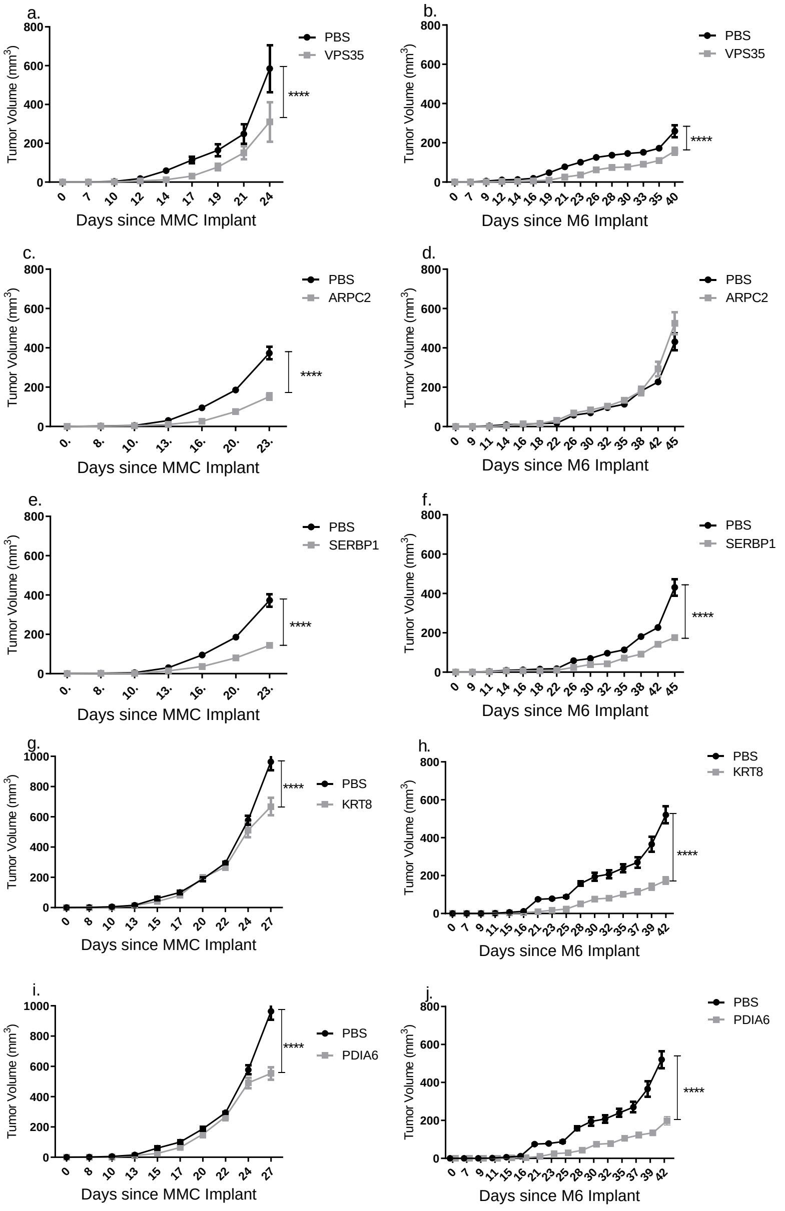

Supplementary Figure 4: Tumor inhibition in implanted tumors of the 5 candidate targets in TgMMTV-neu and C3(1)Tag mice. 8 mice per group were vaccinated with antigen-specific peptides or PBS with CFA/IFA adjuvant (a and b VPS35, c and d ARPC2, e and f SERBP1, g and h KRT8, and I and J PDIA6). a, c, e, g, and i are in TgMMTV-neu mice with MMC implants, b,d, f, h, and j are in C3(1)Tag mice with M6 implants. All comparisons are two-way ANOVA with Bonferroni correction for multiple comparisons. Error bars are standard error of mean (SEM) **** $p<0.0001$

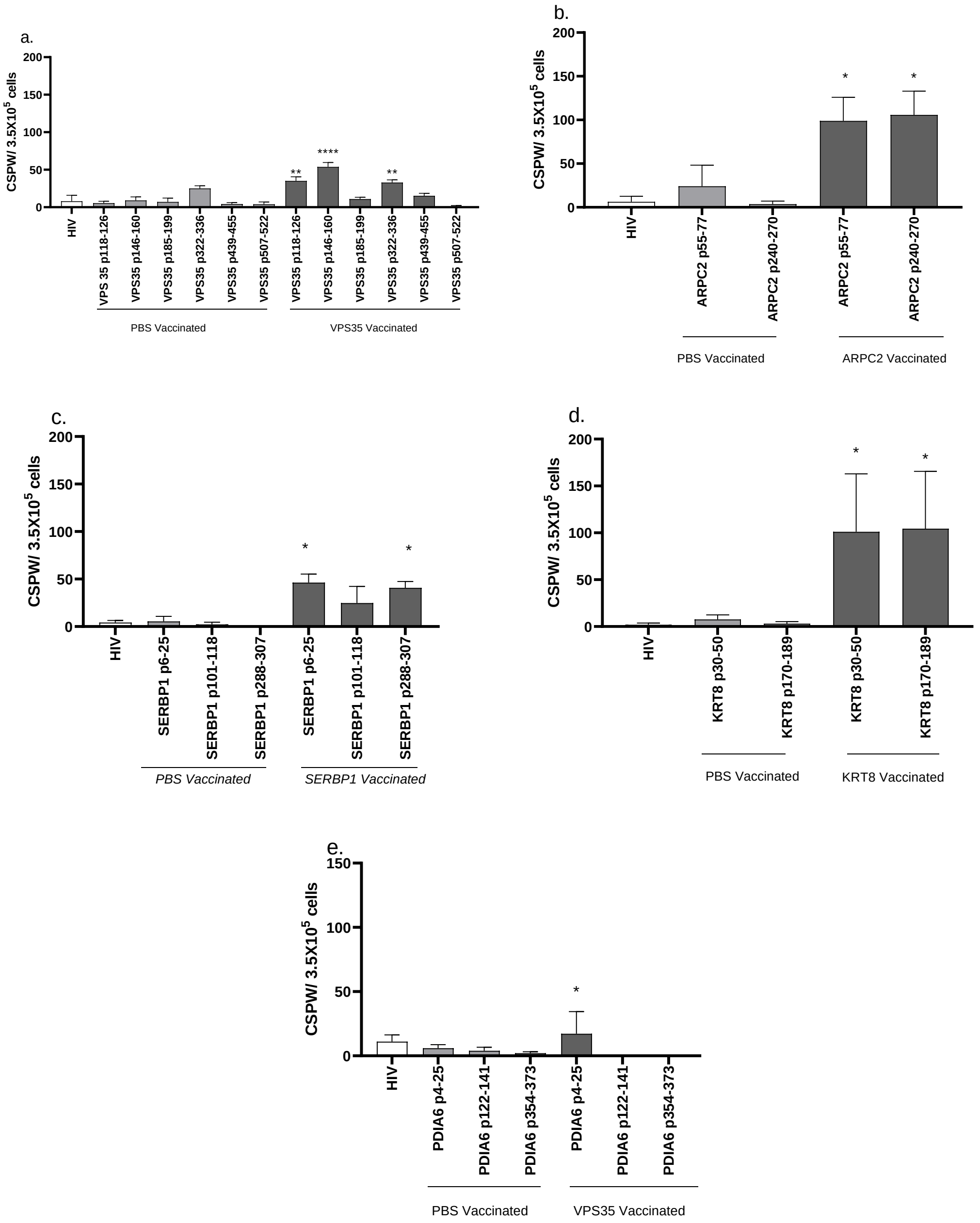

Supplementary Figure 5: IFN-g ELISPOT evaluation from the TgMMTV-neu mice comparing PBS vaccinated (light gray) and antigen-specific peptide (dark gray) in (a) VPS35, (b) ARPC2 (c) SERBP1 (d) KRT8 and (e) PDIA6. Four mice per group were evaluated. The X axis shows the peptides used and the y axis shows average corrected spots per well with $3.5X10^5$ cells/well (corrected for no antigen wells). The p values were calculated in the antigen-vaccinated mice by one-way ANOVA against the HIV negative control well corrected for multiple comparisons by Dunnett's correction. Error bars are standard error of mean (SEM) * p=0.05, ** =0.01 ***p=0.001 **** p<0.0001 .
